# Sensitization of Resistant Breast Cancer Cells with a Jumonji Family Histone Demethylase Inhibitor

**DOI:** 10.3390/cancers14112631

**Published:** 2022-05-26

**Authors:** Balraj Singh, Vanessa N. Sarli, Anthony Lucci

**Affiliations:** 1 Department of Breast Surgical Oncology, The University of Texas MD Anderson Cancer Center, Houston, TX 77030, USA; vnsarli@mdanderson.org; 2 Morgan Welch Inflammatory Breast Cancer Research Program and Clinic, The University of Texas MD Anderson Cancer Center, Houston, TX 77030, USA

**Keywords:** resistant TNBC, intra-tumoral heterogeneity, breast cancer relapse, breast cancer epigenome, metastasis prevention, intrinsic resistance of cancer, tumor adaptability, targeting resistant cancer, quiescent cancer cells, cancer quiescence model

## Abstract

**Simple Summary:**

Using a cell culture model of resistant breast cancer cells with the phenotype that is often responsible for the early relapse of triple-negative breast cancer, namely, the persistence of these cells in reversible quiescence under a variety of challenges, we found that reprogramming the epigenome by treatment with JIB-04, a small-molecule inhibitor of Jumonji-family histone demethylases, sensitized resistant cells. We used this model of deep intrinsic resistance featuring many molecular mechanisms of achieving this phenotype to perform lengthy evaluations of less cytotoxic doses of JIB-04. We found that resistant cells derived from triple-negative inflammatory breast cancer cell lines were either much more sensitive to JIB-04 than the parental cell line or altered by the treatment such that they became sensitive to the chemotherapeutic drugs paclitaxel and doxorubicin. Notably, JIB-04 exposure increased PD-L1 expression in cancer cells, which means that JIB-04 may have clinical applications in improving the responses of triple-negative breast cancer to anti-PD-L1 therapy.

**Abstract:**

In the present study, we evaluated JIB-04, a small-molecule epigenetic inhibitor initially discovered to inhibit cancer growth, to determine its ability to affect deep intrinsic resistance in a breast cancer model. The model was based on a function-based approach to the selection of cancer cells in a cell culture that can survive a variety of challenges in prolonged, but reversible, quiescence. These resistant cancer cells possessed a variety of mechanisms, including modifications of the epigenome and transcriptome, for generating a high degree of cellular heterogeneity. We found that long pretreatment with JIB-04 sensitized resistant triple-negative inflammatory breast cancer cells and their parental cell line SUM149 to the chemotherapeutic drugs doxorubicin and paclitaxel. Resistant cancer cells derived from another inflammatory breast cancer cell line, FC-IBC02, were considerably more sensitive to JIB-04 than the parental cell line. Investigating a mechanism of sensitization, we found that JIB-04 exposure increased the expression of PD-L1 in resistant cells, suggesting that JIB-04 may also sensitize resistant breast cancer cells to anti-PD-L1 immune therapy. Finally, these results support the usefulness of a cell culture-based experimental strategy for evaluating anticancer agents, such as JIB-04, that may halt cancer evolution and prevent the development of cancer resistance to currently used therapies.

## 1. Introduction

Our main study interest is developing therapies for difficult-to-treat breast cancers, such as inflammatory breast cancer (IBC). Breast cancer is divided into three subtypes for alignment with therapy options: estrogen receptor-positive, HER2-positive, and triple-negative (for a lack of estrogen receptor and progesterone receptor, and a lack of HER2 gene amplification) breast cancer. Triple-negative breast cancer (TNBC) is relatively heterogeneous, and, due to a lack of suitable targeted therapies, chemotherapeutic drugs play a major role in the treatment of TNBC. Inflammatory breast cancer, which is much more aggressive than non-IBC, is also categorized into the same three subtypes for treatment. Although the therapies work for IBC to a degree, the outcomes are much worse than those of non-IBC. More recently, immunotherapy involving a checkpoint blockade with anti-PD-L1 antibodies has gained momentum in the treatment of TNBC and TN-IBC. Responses to immunotherapy are thus far limited, and there is a great need to understand the nature of resistance to immunotherapy and investigate ways to overcome it. A recent paper highlighted an important role of quiescent cancer cells in resisting T cell attack in a model of TNBC [1]. Besides other approaches, e.g., modulating the tumor microenvironment, it will be useful to design approaches to inhibit cancer cells that persist in quiescence.

To briefly describe the difficulties in developing therapies for difficult-to-treat cancers, we must first examine the nature of the disease and possible ways of halting its development in a timely manner before it advances to clinical metastasis. Cancer progression follows an evolution-like process, which is shaped by genomic alterations (plus non-genomic alterations) and various selection pressures in the body [2,3,4,5,6]. The deep intrinsic treatment resistance of cancer is governed by a small subpopulation of progenitor-like cancer cells that acquire multiple abnormalities, including not only gene mutations, but also alterations of the epigenome and transcriptome. Significantly, these cells are effective at opportunistically switching to quiescence to survive a variety of challenges in the body, including current therapies that primarily inhibit the growth of or kill actively proliferating cancer cells. Such resistant cells persist in the body as minimal residual disease (MRD) [7,8,9].

In developing an approach for modeling resistance in cell cultures, a crucial difference between the cancer cells in the body and the cancer cells modeled in cancer cell lines lies in the lack of selection pressures on an ongoing basis in cell cultures. This results in the accumulation of cancer cells that would likely not survive in the body, particularly at a time when the tumor burden is very low and the immune system is functioning well, such as the MRD stage before it advances to clinical metastasis. Hypothesizing that a small subpopulation of cancer cells capable of generating a poor-prognosis MRD phenotype may persist in cancer cell lines, even without body-like selection pressures, we chose to apply a body-like metabolic challenge, i.e., a lack of glutamine in culture medium, for selecting such resistant cells [10]. Since the bulk of TN-IBC cells are addicted to glutamine, this selection eliminated approximately 99.99% cells, while selecting rare cells that survived in quiescence for several weeks, and then proliferated indefinitely (referred as SUM149-MA or MA for metabolic adaptability).

SUM149-MA cells are capable of surviving other metabolic challenges, such as a lack of glucose or a lack of oxygen, as well [10,11]. From the therapy perspective, they are resistant to chemotherapeutic drugs and a variety of targeted therapies, such as those targeting the EGFR-MEK/MAPK pathway, and other agents, indicating their pan-resistant nature [12]. Significantly, SUM149-MA cells are highly tumorigenic in nude mice (200 cells injected into fat pads yielding tumors) and they efficiently metastasize to multiple organs, such as the lungs, brain, and skin in nude mice [10]. Their molecular characterization showed a large number of genetic changes (gene amplifications, gene deletions, and mutations) [12]. Gene expression data revealed that they have networks for high epithelial-to-mesenchymal transition (high ZEB1, low GRHL2, and high SNAIL1), which were confirmed by Western blotting [12,13]. They have alterations in alternative splicing (low ESRP1, low ESRP2) that may also be tied to cellular plasticity and the generation of EMT-associated CD44 variant CD44s [14]. Interestingly, SUM149-MA cells carry the FTO gene amplification, which is expressed [15]. FTO is an RNA demethylase, which is associated with obesity. In nature, FTO plays an important role in organismal evolution under conditions of nutrient scarcity. It appears that resistant MA cells can exploit a variety of mechanisms, including those involved in organismal evolution, for their own adaptability and survival under challenges. We interpret that alterations in alternative splicing and base modifications in transcriptome (by FTO and several other regulators) may be a way of generating cellular diversity under a selection pressure. It appears that a lack of glutamine, which forces the selection of an adaptable metabolic state, also co-selects an inter-connected regulatory state [16,17], thus yielding very resistant/adaptable cancer cells. These results point to multiple mechanisms that may complement each other in generating highly resistant and adaptable cancer cells. Overall, these results point to the power of a strong selection pressure in revealing gene networks that are important in cell survival under challenging conditions, which may shape the trajectory of cancer evolution/progression.

The concept of cancer cell adaptability being a driving force in cancer progression is well accepted. However, how to assess cancer cell adaptability in cell cultures is not obvious. Since cell adaptability has many characteristics, first, we must identify which characteristics we want to assess. A characteristic that is appealing in this regard is an opportunistic switching of cancer cells between quiescence and proliferation, which has clinical relevance at the MRD stage. This is also a definable characteristic in cell culture when cells are subjected to a challenge, such as a metabolic challenge or a therapeutic challenge. However, assessing this phenotype in cell cultures is complicated because we have to distinguish a relatively small number of cells with this phenotype from the bulk of cells that simply proliferate in cell cultures without having this adaptability phenotype. Simply having selected MA cells under a metabolic challenge is a major first step, but it does not eliminate the shortcomings of cell culture, since robust cell proliferation is a common ongoing feature in cell cultures when cells are not under a challenge. Our solution to this vexing problem is to utilize existing methods in a way that increases the attention towards the rare, but most-adaptable, cells. The core element of the approach behind the selection of MA cells and how a therapy would affect adaptable cancer cells is based on a reasonable assumption that most cells proliferating in cell cultures are relatively sensitive to currently offered therapies. Therefore, we allow sufficient time for therapeutic agents to kill approximately 99% of cells, and then assess what is left as resistant cells with desirable adaptability characteristics. This approach reveals whether a therapeutic agent has the potential for overcoming deep intrinsic resistance, such as the one seen in poor-prognosis MRD. In essence, our approach is designed to reduce the “noise” created in cell culture due to (1) a lack of body-like challenges on an ongoing basis, and (2) the artificiality of the cell culture system, which is typically developed to optimize cell proliferation at all costs.

TN-IBC-derived MA cell lines yield adaptable cancer cells that are very robust in switching back and forth between quiescence and proliferation, which validates this approach to modeling deep intrinsic resistance. In contrast, non-IBC triple-negative breast cancer cell lines, such as MB-MDA-231 and their metastatic variants, selected from two rounds of bone metastases in nude mice after the cardiac inoculation of cancer cells yielded cells that could persist in quiescence but failed to grow as healthy cell cultures for an indefinite period [10]. This phenotypic difference between IBC and non-IBC cells, considered in the context of cancer progression, explains the basis for early, rather than late, relapse of IBC [18,19].

In the present study, we evaluated the ability of JIB-04, a pan-inhibitor of Jumonji-family histone demethylases, to affect intrinsic resistance in our model of adaptable TN-IBC cells. Wang et al. identified JIB-04 as an epigenetic modulator in a cancer-selective manner using a cell-based screen [20]. JIB-04 inhibits cancer cell growth in vitro and in vivo. It also has increased survival durations in mice bearing aggressive 4T1 breast cancer xenografts [20]. Subsequent studies showed that JIB-04 inhibited resistant cancer cells for several cancer types [21,22,23,24,25,26,27], making it a desirable small-molecule inhibitor. We found that resistant cancer cells were more sensitive to JIB-04 than the parental cell line was, and that long treatment with JIB-04 sensitized the resistant cancer cells to chemotherapeutic drugs, demonstrating the clinical potential of this inhibitor.

## 2. Materials and Methods

### 2.1. Cell Lines and Drugs

The resistant TN-IBC cell line used in this study was SUM149-MA, which was derived from a firefly luciferase-transfected SUM149 cell line (SUM149-Luc) [28]. We obtained the SUM149 cell line from Stephen Ethier (then at Barbara Ann Karmanos Cancer Institute, Detroit, MI, USA). The resistant TN-IBC cell line FC-IBC02-MA, which was derived from the FC-IBC02 cell line [29], was also used. The generation and characterization of these cell lines and their culture conditions were described previously [10,11]. Because the selection of MA cell lines was performed using a medium lacking glutamine, dialyzed fetal bovine serum (FBS) was used instead of regular FBS to further reduce the glutamine levels in the culture medium. Therefore, all experiments were performed using media containing dialyzed FBS for consistency, even when the medium was supplemented with glutamine (parental cell lines and MA cell lines after the initial selection in the absence of glutamine).

We purchased JIB-04 from Selleck Chemicals (Houston, TX, USA), and paclitaxel and doxorubicin from Sigma-Aldrich (St. Louis, MO, USA). We dissolved JIB-04, paclitaxel, and doxorubicin in dimethyl sulfoxide (DMSO). We added equal volumes of DMSO without drugs to the cultures of the control dishes. The DMSO volume was equal to or less than 0.04% of the volume of the culture medium.

### 2.2. Western Blotting

We performed Western blotting to detect protein bands as enhanced chemiluminescence signals on x-ray films as described previously [30]. We used anti-PD-L1 (catalog number 13684; Cell Signaling Technology, Danvers, MA, USA) and anti-HSP90 (catalog number 4875; Cell Signaling Technology) antibodies for protein detection. After the detection of PD-L1, the Western blot membranes were re-probed to detect HSP90, which served as an internal control for the normalization of protein loading. Each Western blot was performed at least twice. We quantified the relative intensities of the protein bands detected on x-ray films with ImageJ software (version 153; National Institutes of Health, Bethesda, MD, USA).

### 2.3. JIB-04 Treatment of Resistant Cancer Cells

Resistant and parental TN-IBC cells were treated in parallel with JIB-04 at 62.5–250.0 nM for various periods. The effects of this treatment manifested in two different ways: (1) massive cell killing followed by the growth of colonies in the presence of JIB-04 and (2) massive cell killing with the requirement of the removal of JIB-04 for the recovery of the remaining viable cells and their growth into colonies. The effects of these treatments on cell growth and morphology were monitored frequently under a microscope. At the end of treatment, the culture dishes containing the colonies were stained with crystal violet and photographed or scanned. For the quantitation of the relative cell masses in the stained dishes, the colonies were counted.

### 2.4. Assay of Relative Resistance of Cells to Paclitaxel and Doxorubicin

To determine whether treatment with JIB-04 affected the sensitivity of the resistant TN-IBC cells to the chemotherapeutic drugs, we allowed drug-treated cells to recover for a few days and then passaged them. These JIB-04-treated cells were treated in parallel with control vehicle-treated cells for 5–8 days with predetermined concentrations of chemotherapeutic drugs (5 nM paclitaxel and 100 nM doxorubicin) that killed 99% of the proliferating cells. We then removed the chemotherapeutic drugs by changing the medium and let the surviving cells form colonies for 10–12 days. We stained the colonies with crystal violet and counted them.

## 3. Results

### 3.1. JIB-04 Resistance of SUM149-MA Cells

**Figure 1:**
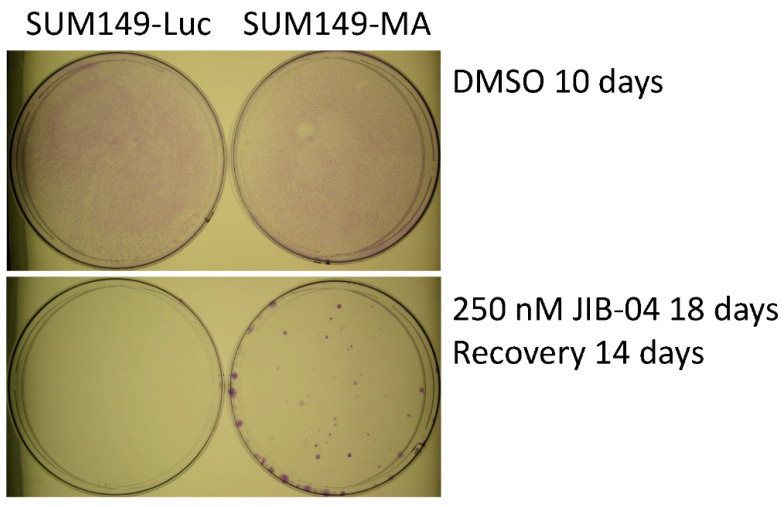
Resistance of SUM149-MA cells to treatment with JIB-04. SUM149-MA and parental SUM149-Luc cells were treated in parallel with 250 nM JIB-04 for 18 days (treatment killed most of the cells) and then allowed to recover and grow into colonies in a drug-free medium for 14 days before staining the colonies (**bottom**). The (**top**) panel shows the input control dishes from the treatment of cell cultures with the solvent DMSO for 10 days, which was when both cell cultures grew to comparable confluency as indicated by crystal violet staining.

To evaluate the resistance to JIB-04 of the SUM149-MA cells relative to that of parental SUM149-Luc cells, we treated cultures of both cell lines with different concentrations of JIB-04 and observed them under a microscope. We changed the drug medium as needed to remove floating dead cells from the culture dishes. Typically, when most of the cells were killed and about 1% of the cells were still attached to the dishes, we shifted the cells to fresh medium without JIB-04 and let the resistant cells grow into colonies to evaluate the relative resistance of these cells to JIB-04 in both cultures. In these experiments, we consistently observed considerably more colonies in the SUM149-MA cultures than in the SUM149-Luc control cultures. Figure 1 shows the results of a representative experiment in which we treated SUM149-MA and SUM149-Luc cells with 250 nM of JIB-04 for 18 days and then allowed the remaining resistant cells to recover and grow into colonies in the absence of JIB-04 for 14 days. The figure shows about 50 SUM149-MA cell colonies of various sizes; the culture of control parental SUM149-Luc cells treated with JIB-4 in parallel did not yield any colonies. This result is similar to the results obtained for most agents, including the chemotherapeutic drugs doxorubicin and paclitaxel, demonstrating the highly adaptable and resistant nature of these cells [10,11,12,13,14,15].

### 3.2. Sensitization of SUM149-MA Cells to Chemotherapeutic Drugs by Treatment with JIB-04

**Figure 2:**
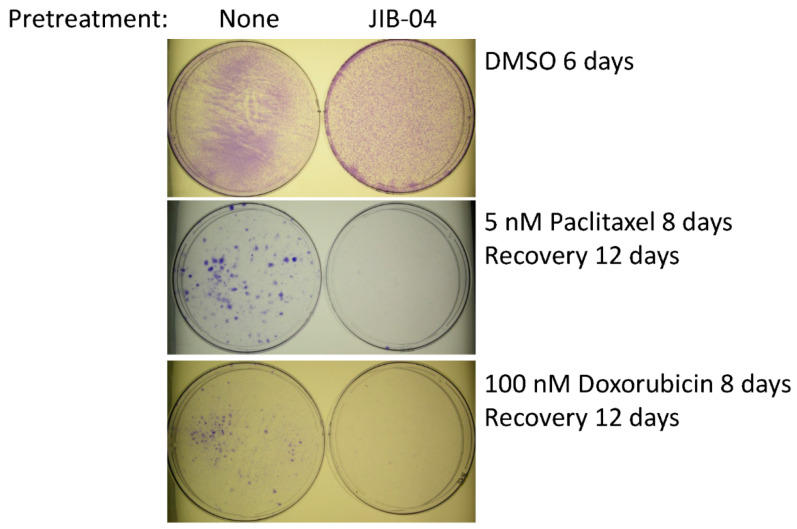
Treatment with JIB-04 sensitizes SUM149-MA cells to treatment with chemotherapeutic drugs. After a 10-day treatment with 125 nM JIB-04, we allowed surviving cells to recover in a drug-free medium for 7 days. Following this, we trypsinized the cells, plated them into fresh culture dishes, treated them in parallel with 5 nM paclitaxel (**middle** panel) or 100 nM doxorubicin (**lower** panel) for 8 days, and then let them recover and grow into colonies for 12 days before staining with crystal violet. Cells treated with DMSO in parallel that were stained 6 days later served as controls (**top** panel). Representative images of cell cultures taken at 10× magnification are shown. These images show that pretreatment with JIB-04 significantly decreased the number of colonies.

Next, we asked whether treatment with JIB-04 alters the resistance of TN-IBC cells according to useful criteria (e.g., their sensitivity to chemotherapeutic drugs), even though they are not killed during *in vitro* treatment. To answer this question, we pretreated SUM149-MA cells with JIB-04, allowed them to recover and grow in the absence of JIB-04, and then tested their sensitivity to treatment with doxorubicin or paclitaxel as compared with that of control MA cells not pretreated with JIB-04. Figure 2 shows the representative results of the pretreatment of SUM149-MA cells with 125 nM JIB-04 for 10 days, followed by recovery and growth for 7 days. We then evaluated their relative resistance to chemotherapeutic drugs, which involved an 8-day treatment with either 5 nM paclitaxel or 100 nM doxorubicin (approximately only 1% of morphologically abnormal cells in total survived these treatments) followed by the recovery and growth of resistant cells into colonies for 12 days. We observed many colonies (more than 200) of different sizes of the control SUM149-MA cells not pretreated with JIB-04. In contrast, SUM149-MA cells pretreated with JIB-04 exhibited a dramatic reduction in the number of colonies after treatment with either paclitaxel or doxorubicin, with the observation of only a few small colonies (fewer than five). These findings demonstrated that treatment with JIB-04 modulated the epigenetic state of resistant cancer cells toward a favorable therapy-sensitive state.

**Figure 3:**
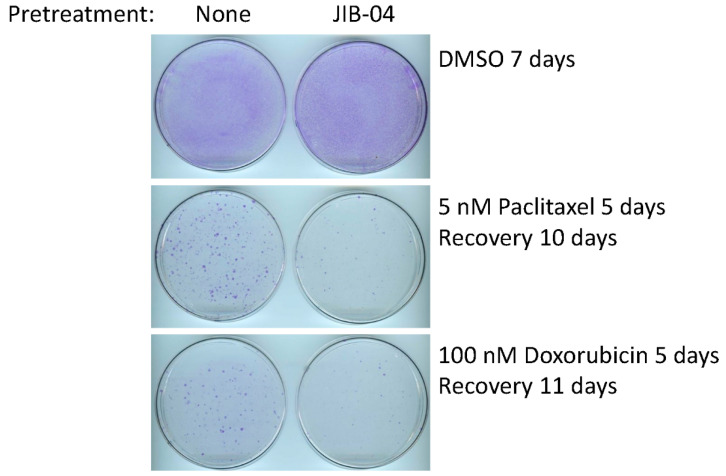
Pretreatment with JIB-04 sensitizes SUM149-Luc cells to chemotherapeutic drugs. After a 10-day treatment with 125 nM JIB-04, we allowed the surviving cells to recover in a drug-free medium for 15 days, trypsinized them, and plated them into fresh culture dishes. Following this, we treated the cells in parallel with 5 nM of paclitaxel (**middle** panel) or 100 nM of doxorubicin (**bottom** panel) for 5 days and then let them recover and grow into colonies for 10 or 11 days, as indicated before staining with crystal violet. Cells treated with DMSO in parallel and stained 7 days later served as controls (**top** panel). These images show that pretreatment with JIB-04 significantly decreased the number of colonies.

Although SUM149-MA cells are highly adaptable and resistant [10,11,12,13,14,15], we recognize that the parental cell line SUM149 is one of the most resistant TNBC cell lines. Therefore, determining whether treatment with JIB-04 also sensitizes parental SUM149 cells to chemotherapeutic drugs is useful. To that end, we pretreated SUM149-Luc cells with JIB-04 and then tested their sensitivity to paclitaxel and doxorubicin. Specifically, we pretreated the cells with 125 nM of JIB-04 for 10 days, allowed them to recover and grow for 15 days, and then treated them with 5 nM of paclitaxel (5 days followed by 10 days of recovery) or 100 nM of doxorubicin (5 days followed by 11 days of recovery). Based on the microscopic examination after treatment with chemotherapeutic drugs (prior to recovery), we observed that the treatments eradicated more than 99% of the cells, leaving behind only a few morphologically abnormal cells in total. We found that the pretreatment resulted in dramatically fewer colonies than the control cells that were not pretreated with JIB-04, demonstrating that the pretreatment sensitized SUM149-Luc cells to both chemotherapeutic drugs equally (Figure 3).

Since JIB-04 inhibits the parental cell line more than it does MA cells, it became necessary to allow a longer recovery time for us to have sufficient number of cells for assessing the response to chemotherapeutic drugs of the remaining cells from the parental cell line. In these experiments, the objective was to determine whether JIB-4 treatment sensitized both cell lines. Since MA cells are much more resistant to chemotherapeutic drugs, they can handle longer treatments that parental cells cannot. The objective was to compare JIB-04-treated cells with untreated cells, not compare parental cells with MA cells. Therefore, we adjusted the treatment times to kill about 99% of the cells, which revealed the most-resistant cancer cells after their recovery.

### 3.3. Increased PD-L1 Expression in SUM149-MA Cells upon Treatment with JIB-04

**Figure 4:**
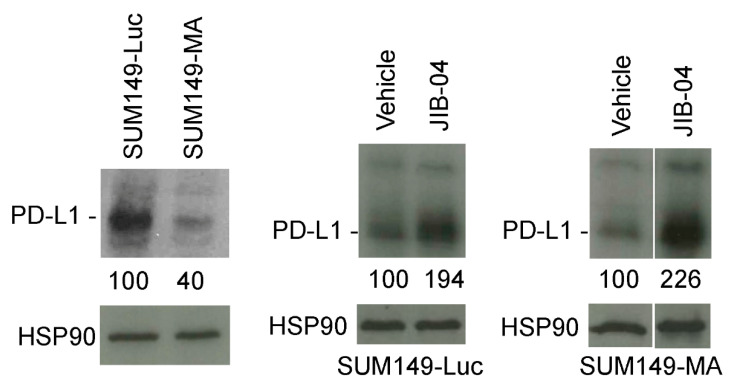
Increased PD-L1 protein levels in TN-IBC cells upon treatment with JIB-04. (**Left**) The PD-L1 protein level in SUM149-MA cells was lower than that in the parental SUM149-Luc cell line. The relative levels of PD-L1 protein in SUM149-MA and parental SUM149-Luc cells cultured in glutamine-containing medium with dialyzed FBS were determined via Western blotting. SUM149-Luc (**middle**) and SUM149-MA (**right**) cells were treated with 62.5 nM JIB-04 for 10 days, allowed to recover for 10 days in a drug-free medium, and subjected to Western blotting to compare the PD-L1 protein levels. Nitrocellulose membranes were re-probed with an anti-HSP90 antibody to normalize sample loading. The relative levels of PD-L1 protein, as quantitated by ImageJ analyses of band intensities, are shown below the PD-L1 blots. Cropped western blots are shown; see Figure S1 for uncropped blots.

Our analysis of the gene expression data showed that SUM149-MA cells had reduced PD-L1 mRNA expression [12]. Considering that anti-PD-L1 antibodies are increasingly used in the treatment of TNBC, we decided to validate the gene expression data with Western blotting. We found that SUM149-MA cells had lower (by 60%) PD-L1 expression than parental SUM149-Luc cells (Figure 4, left). Our interpretation of this finding is that the lower PD-L1 expression in SUM149-MA cells may result from their progenitor cell-like nature. Studies have demonstrated that TNBC cells that express PD-L1 may be more sensitive than cells not expressing it to therapies in general, not just anti-PD-L1 antibodies. A clinical trial that demonstrated a markedly better pathologic complete response of PD-L1–positive TNBC than PD-L1–negative TNBC to neoadjuvant therapy [31] supports this assertion. To assess the potential clinical relevance of PD-L1 expression, we sought to determine whether treatment with JIB-04 increased PD-L1 expression in SUM149-MA cells as part of the reprogramming of the epigenome toward a “differentiated state” that may be a feature of relatively therapy-sensitive TNBC cells. Western blot analyses showed that a 10-day treatment with 62.5 nM JIB-04 (followed by a 10-day recovery period) significantly increased (2.26-fold) the level of PD-L1 protein in SUM149-MA cells (Figure 4, right). A similar treatment with JIB-04 also significantly increased (1.94-fold) the PD-L1 protein levels in parental SUM149-Luc cells (Figure 4, middle). For these experiments we used a 62.5 mM dose of JIB-04, which was tolerated better by both cell lines. This allowed us to treat both cell lines similarly with a treatment time of 10 days and recovery period of 10 days. We noted that the fold increase in the PD-L1 protein level in these cells over that in the untreated control cells was higher in the SUM149-MA cells than in the parental SUM149-Luc cells, demonstrating that resistant cells may be more sensitive to treatment with JIB-04 in this regard.

### 3.4. Sensitivity of FC-IBC02-MA Cells to Treatment with JIB-04

**Figure 5:**
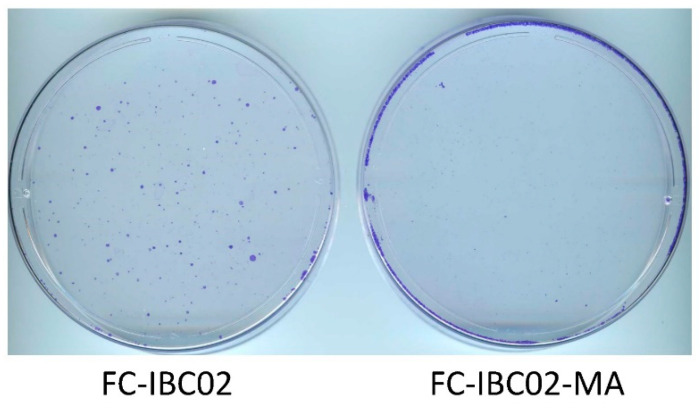
Inhibition of the growth of FC-IBC02 and FC-IBC02-MA cells by treatment with JIB-04. Cultures of both cell lines were treated in parallel with 62.5 nM JIB-04 for 14 days and then stained with crystal violet. Based on a comparison of the number of colonies in the images, JIB-04 affected the FC-IBC02-MA cells much more severely than the parental FC-IBC02 cells.

Next, we evaluated JIB-04 in another resistant TN-IBC cell line model that we recently developed, namely, adaptable FC-IBC02-MA cells derived from the FC-IBC02 cell line [11]. We found that a 14-day treatment with 62.5 nM JIB-04 killed most of the cells in the culture. However, a small number of cells survived and grew into colonies during this period. A comparison of FC-IBC02-MA and FC-IBC02 cells showed that the parental FC-IBC02 cells yielded a significant number of colonies, whereas the FC-IBC02-MA cells yielded only a few colonies (Figure 5). This significant difference in the number of colonies suggested that resistant cells had increased sensitivity to JIB-04. This result, along with the results described above for SUM149-MA cells, supports the idea, which is also backed by several published studies on different cancers, that JIB-04 may be a suitable agent for targeting resistant cancer cells in patients with poor-prognosis cancers [21,22,23,24,25,26,27].

## 4. Discussion

Using a novel cell culture model of the deep intrinsic treatment resistance of cancer, which allows for therapeutic evaluation in rare cancer cells that can persist in reversible quiescence under a variety of pressures, we showed that treatment with low-dose JIB-04 may inhibit the progression of breast cancer. Concerning how treatment with JIB-04 may sensitize resistant cancer cells, the modulation of the epigenome may shift the cell fate from stem-like to non-stem-like. However, we must remember that, analogous to the persistence of a pool of stem-like cancer cells in the body, such a pool also likely persists in our system under treatment with JIB-04. We hypothesize that, although treatment with JIB-04 does not completely eradicate resistant cancer cells, it changes them enough that it may provide a clinical benefit. Our results demonstrated that JIB-04 may go deeper under intrinsic resistance than other therapies that mainly target proliferative cancer cells.

Let us briefly describe the rationale for the design of assays for assessing intrinsic resistance in cell cultures. Most proliferating cancer cells in cell cultures are not adaptable and they are very sensitive to therapies. Our interest lies in assessing a phenotype similar to poor-prognosis MRD in cancer patients, i.e., an ability of cancer cells to survive in quiescence under all challenges, including chemotherapeutic agents. MA cells (0.01% in cell culture) with this phenotype have been isolated under a metabolic challenge; however, in the absence of a selection pressure in the cell culture, there would be a tendency to shift the equilibrium toward a proliferative/sensitive state. For this reason, our assays typically involve the elimination of proliferating cells under treatment with a drug, leaving behind rare cells that can survive in quiescence. At this point, we remove the drug and let the surviving cells gradually grow into colonies, which we visualize by staining with crystal violet. In colony formation assays, our purpose is to simply ask whether the rare cells on the dish can proliferate. In this format, we can follow the cells in the culture for long periods as needed (some cells start proliferating sooner than others), and also assay the agents that would extend the dormancy period in resistant cancer cells.

Although we did not investigate which specific histone demethylases are the targets of JIB-04 in our model, two specific Jumonji histone demethylases were of interest. Specifically, our previously published gene expression and gene copy number data demonstrated that JMJD1B/KDM3B was amplified and JHDM1D/KDM7A was overexpressed in SUM149-MA cells [12]. Of note is that both of these demethylases were also overexpressed in paclitaxel- and carboplatin-resistant lung cancers, and that resistant cells were hypersensitive to JIB-04 treatment in preclinical models of lung cancer [23]. These results raise the possibility that KDM3B and KDM7A are targets of JIB-04 in resistant TNBCs, such as TN-IBC, which can be investigated in the future.

If validated further in preclinical animal studies, epigenetic modulators, such as JIB-04, may be suitable for evaluation in clinical trials to determine their potential to inhibit relapse in high-risk patients, such as those with TN-IBC. As to how JIB-04 could be useful in the setting of immune checkpoint blockade therapy for TNBC, it is worth mentioning that, in some studies, anti-PD-L1 antibodies were offered to TNBC patients based on their PD-L1 positivity, as they increase the likelihood of response [32]. Other studies have not shown a response dependent on PD-L1, possibly due to the use of different drugs, different disease stages (early versus late), and/or PD-L1 assays utilized [33]. In any case, if treatment with JIB-04 alters TNBC cells similarly to what we observed in the cell culture (i.e., increases their PD-L1 expression), a useful next step would be to determine whether pretreatment with JIB-04 converts the PD-L1 status from negative to positive in mouse models and patients with high-risk TNBC, thus converting anti-PD-L1 therapy non-responders to responders.

## 5. Conclusions

Based on our results described herein, we concluded that JIB-04 is a suitable agent for overcoming therapy resistance in cancers such as TN-IBC. We obtained our results using a cell culture model of deep intrinsic resistance. Others have reported similar findings for several cancer types using different experimental models, specifically, cell lines and xenograft tumor models in mice. Whether JIB-04 or compounds similar to it will advance to clinical trials remains to be seen.

Besides providing useful information regarding the potential of JIB-04 to overcome therapy-resistant IBC, this study further validates our cell culture-based model of deep intrinsic resistance as a useful addition to other models that guide the development of cancer therapy. The application of this model with various cancers has the potential to expedite therapy development and thus improve outcomes.

## Data Availability

The data presented in this study are available in this article (and Supplementary Material).

## Supplementary Materials

The following are available online at https://www.mdpi.com/article/10.3390/cancers14112631/s1, Figure S1: Uncropped Western blots used for Figure 4.

## Abbreviations

6-MP: 6-mercaptopurine; DMSO: dimethyl sulfoxide; FBS: fetal bovine serum; IBC: inflammatory breast cancer; TNBC: triple-negative breast cancer; TN-IBC: triple-negative inflammatory breast cancer; MA: metabolically adaptable.
