# Supplementary material for: Sensitization of Resistant Breast Cancer Cells with a Jumonji Family Histone Demethylase Inhibitor
Source: Cancers (Basel). 2022 May 26;14(11):2631. doi: 10.3390/cancers14112631 (PMC9179491; doi:10.3390/cancers14112631)
Supplement: Supplementary file 1 [file cancers-14-02631-s001.zip › cancers-1707385-supplementary.pdf]

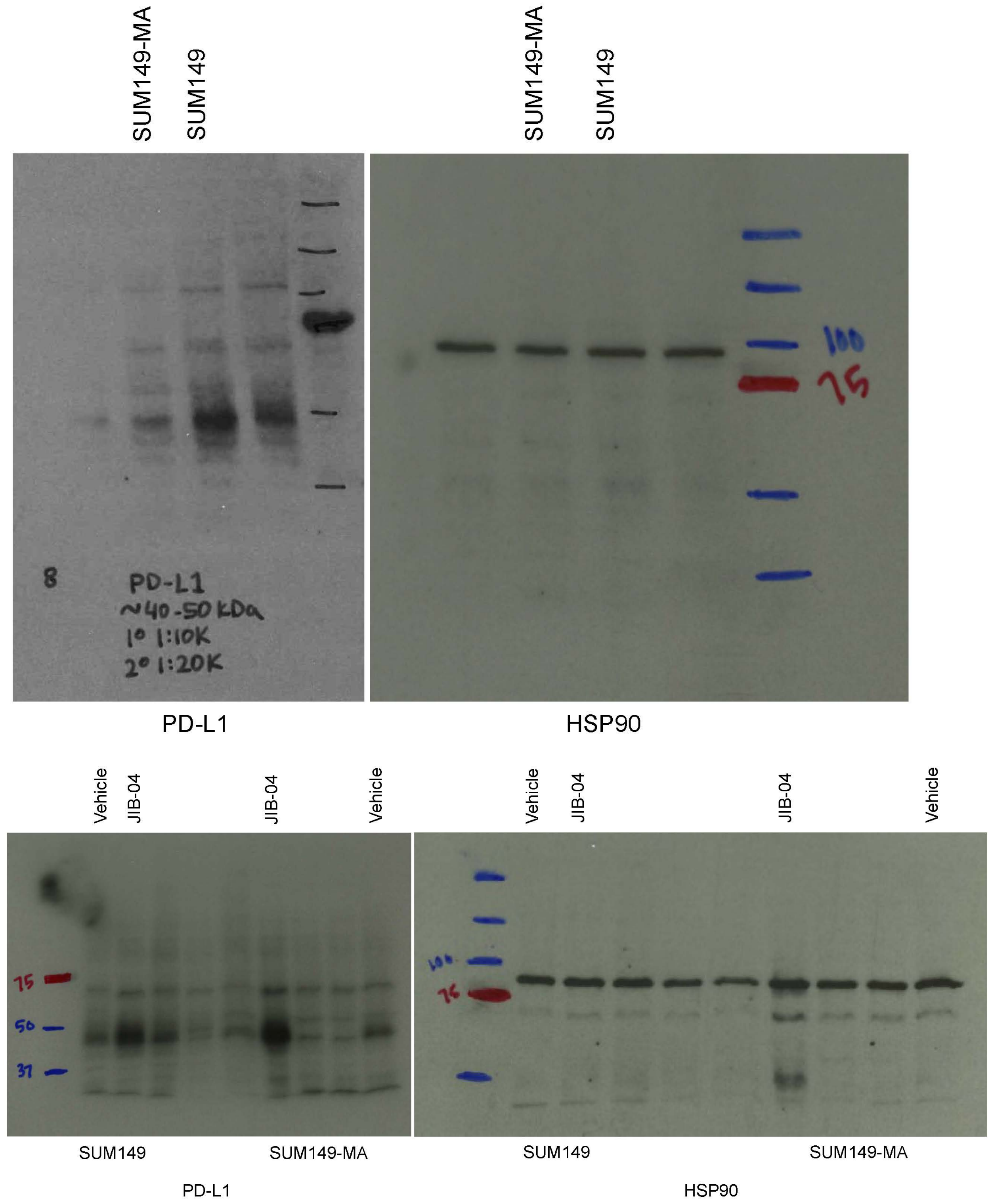

Supplementary Figure S1. Uncropped Western blots used for Figure 4. The blots on the top are for left panel of Figure 4. The blots at the bottom are for middle and right panels of Figure 4. The lanes used in Figure 4 are labeled, and cell lines and JIB-04-treated lanes are identified.
